# Fertility trends during successive novel infectious disease outbreaks: Zika and COVID-19 in Brazil

**DOI:** 10.1590/0102-311XEN230621

**Published:** 2022-04-29

**Authors:** Leticia Junqueira Marteleto, Luiz Gustavo Fernandes Sereno, Raquel Zanatta Coutinho, Molly Dondero, Sandra Valongueiro Alves, Ryan Lloyd, Andrew Koepp

**Affiliations:** 1 University of Texas at Austin, Austin, U.S.A.; 2 Faculdade de Ciências Econômicas, Universidade Estadual de Campinas, Campinas, Brasil.; 3 Universidade Federal de Minas Gerais, Belo Horizonte, Brasil.; 4 American University, Washington DC, U.S.A.; 5 Universidade Federal de Pernambuco, Recife, Brasil.

**Keywords:** Fertiliy, Live Birth, COVID-19, Zika Virus, Population Estimates, Fecundidade, Nascido Vivo, COVID-19, Zika Vírus, Estimativas de População, Fecundidad, Nacimiento Vivo, COVID-19, Virus Zika, Estimativas de Población

## Abstract

This study aims to estimate fertility trends in Brazil in the 2010s and early 2020s during a period of back-to-back novel infectious disease outbreaks – Zika virus and COVID-19. We use Brazilian Ministry of Health and Association of Civil Registrar data from 2011–2021 to measure general fertility rates at the national and state levels. We also used seasonal ARIMA model to forecast fertility rates by month and state in 2021 and compared these forecasts with observed fertility rates. We find that fertility rates were steady over 2011–2015 with no statistically significant variation, followed by a sharp decline during the Zika outbreak in 2016 followed by a return to pre-Zika levels after the end of the epidemic. Furthermore, to evaluate the effect of the COVID-19 pandemic, we make comparisons with observed and forecast rates from 2020–2021, showing that declines were generally larger for observed than for forecast rates, yet statistically insignificant. We argue that the resurgence of the COVID-19 pandemic in 2021 might lead to further declines, as women might have not had enough time to adjust rebound from either the effects of the Zika epidemic. We also discuss the importance of timely availability of live births data during a public health crisis with immediate consequences for fertility rates.

## Abstract (pt)

O objetivo desta contribuição de dados é estimar as tendências de fecundidade no Brasil nos anos 2010 e início dos anos 2020 durante epidemias consecutivas de doenças infecciosas novas, ou seja, Zika vírus e COVID-19. Utilizamos dados do Ministério da Saúde e do Registro Civil Nacional de 2011–2021 para calcular as taxas mensais de fecundidade nos níveis nacional e estadual. Também utilizamos o modelo ARIMA sazonal para prever taxas de fecundidade por mês e por estado em 2021, e comparamos essas previsões com as taxas de fecundidade observadas. Encontramos que as taxas de fecundidade eram estáveis entre 2011 e 2015, sem variação significativa, seguido por um declínio abrupto durante o surto de Zika em 2016, e seguido por sua vez por um retorno aos níveis pré-Zika depois do fim da epidemia. Além disso, para avaliar o efeito da pandemia de COVID-19, comparamos as taxas observadas e previstas de 2020–2021, mostrando que as quedas geralmente eram maiores nas taxas observadas do que nas previstas, porém sem significância estatística. Argumentamos que o recrudescimento da pandemia de COVID-19 em 2021 poderá levar a mais quedas nas taxas, na medida em que as mulheres não tenham tido tempo suficiente para reagir e se ajustarem aos efeitos da epidemia de Zika. Também discutimos a importância da disponibilidade oportuna de dados sobre nascidos vivos durante uma crise de saúde pública com consequências imediatas para as taxas de fecundidade.

## Abstract (es)

El objetivo de esta aportación de datos es estimar las tendencias de fecundidad en Brasil en la década de 2010 y principios de 2020, durante el período de brotes consecutivos de nuevas enfermedades infecciosas -ZIKV y COVID-19. Se usaron datos procedentes del Ministerio de Salud y del Registro Civil Nacional (ARPEN) desde 2011–2021 para calcular mensualmente las tasas de fecundidad en nivel nacional y en el estado. Se utilizó el ARIMA estacional para pronosticar las tasas de fecundidad por mes y estado en 2021, y se compararon estas predicciones con las tasas de fecundidad observadas. Encontramos que las tasas de fecundidad se mantuvieron estables entre 2011 y 2015, sin variaciones significativas, seguido de un fuerte descenso durante el brote de Zika en 2016, para posteriormente volver a los niveles anteriores al Zika tras el fin de la epidemia. Asimismo, con el fin de evaluar el efecto de la pandemia de COVID-19, hicimos comparaciones con lo observado y la previsión de tasas desde 2020–2021, que muestran que los descensos fueron en general mayores para los índices observados que para los previstos, aunque insignificantes desde el punto de vista estadístico. Sostenemos que el resurgimiento de la pandemia de COVID-19 en 2021 podría provocar nuevos descensos, ya que las mujeres podrían no haber tenido suficiente tiempo para adaptarse a los efectos de la epidemia de Zika. También se discute la importancia de disponer a tiempo de los datos de los nacidos vivos durante una crisis de salud pública con consecuencias inmediatas para las tasas de fecundidad.

## Introduction

In March 2020, less than three years after the end of the Zika epidemic, Brazil was hit by COVID-19. Brazil is currently an epicenter of the pandemic, with more than 613,642 deaths associated to COVID-19 by the time of writing ^1^.

Brazil was also the epicenter of the Zika epidemic and an accompanying surge in congenital Zika syndrome (CZS) in 2015–2017. Fertility rates declined during the Zika epidemic, with abrupt declines occurring roughly nine months after the association between Zika and fetal malformation was publicized ^2,3^.

Novel infectious diseases, such as COVID-19 and Zika, generate extreme uncertainty about infection risks as well as confusion about prevention, especially regarding typically high-risk groups, such as pregnant women and their infants.

In May 2020, the U.S. Centers for Disease Control and Prevention (CDC) added pregnancy to the list of conditions that made COVID-19 patients more likely to be admitted to intensive care ^4^. Increases in stillbirth and preterm childbirth have been recorded, although it is uncertain whether these increases are the direct results of SARS-CoV-2 infections or the indirect effects of stress and a reluctance to seek medical care ^5^. Evidence from Brazil also shows higher mortality rates for pregnant and post-partum women ^6,7^ and a large number of newborn deaths ^7^. Confronted with the uncertainty surrounding COVID-19, women at risk of pregnancy could avoid childbearing, leading to fertility decreases similar to the ones observed during Zika ^2,3,8,9^.

This analysis aims to examine fertility trends in Brazil during the 2010s and early 2020s, which encompasses the time periods before, during, and after the Zika virus (ZIKV) epidemic, the first wave of the COVID-19 pandemic, and the subsequent economic and political crises. In addition to causing a considerable amount of apprehension, the back-to-back timing of the ZIKV and COVID-19 crises could have also led to further declines in birth rates due to a lack of time for recovery between crises.

## Methods

The general fertility rates (GFR) were calculated to examine fertility trends in Brazil:

$${\text{GFR}}_{ti}=\frac{live\;births_{ti}}{pop\;w\;15-49_{ti}}$$

where *pop w* 15–49_ti_ is the number of women aged 15–49 in each month; *t* represents the year and *i*, the state. The denominators were derived from Bayesian population projections 10. The *live births*_*ti*_ term is the number of births in each month; *t* represents the year and *i*, the state.

To determine live births, we use two publicly available datasets on births. For the 2011–2019 period, we use data from the Brazilian Information System on Live Births (SINASC) from Brazil’s Ministry of Health, with a documented 96% coverage of all births ^11^. Due to SINASC data are still preliminary for 2020 and 2021, we also use data from the Association of Civil Registrar (ARPEN) database, which compiles birth certificates from civil registration offices and is the only finalized publicly available dataset in 2020 and 2021 births in Brazil ^12^. To the best of our knowledge, no study has ever compared birth trends for the post-ZIKV and COVID-19 periods with both datasets, despite the clear need for timely birth-rate analysis during public health crises ^13,14^.

Since the final SINASC and ARPEN datasets overlap for a five-year period, while the SINASC data have a wide coverage of births, we employ three techniques to determine the states in which the ARPEN data are most consistent with SINASC data. Firstly, we confirm that the datasets have been remarkably similar since 2018 (Supplementary Material 1: http://cadernos.ensp.fiocruz.br/static//arquivo/supl-e00230621-1_4278.pdf). We then perform a cointegration test between births in both time series. To do so, we have regressed ARPEN against SINASC birth time series separately for all states. To be cointegrated, the first difference of the series must be stationary (Augmented Dickey Fuller – ADF) test at 5%) and the residuals must be stationary (ADF test at 5%). No sign of residual autocorrelation is necessary: Durbin-Watson (DW) test at 5% and residual lagged coefficient must be not significant (at 5%) ^15^ (Supplementary Material 2: http://cadernos.ensp.fiocruz.br/static//arquivo/supl-e00230621-2_7760.pdf). Regarding explanatory power, the ARPEN births estimated coefficient must be significant at 5% level and its variation must explain at least 50% of the variation from SINASC births (i.e., R2 must be at least 0.5). Finally, we identified states in which ARPEN births are reliable predictors of SINASC births (Supplementary Material 2: http://cadernos.ensp.fiocruz.br/static//arquivo/supl-e00230621-2_7760.pdf). Supplementary Material 3 (http://cadernos.ensp.fiocruz.br/static//arquivo/supl-e00230621-3_7933.pdf) shows the GFRs for the 13 states that meet the criteria for explanatory power and a lack of autocorrelation. These states contained 71.34% of the Brazilian population in December 2019.

Then, seasonal autoregressive integrated moving average (ARIMA) ^16^ was used to forecast 2021 GFRs by month and state using ARPEN data (2018–2020), as well as to forecast 2020 GFRs using SINASC (2011–2019) data. The models were selected using the auto.arima function of the forecast package in the R language (http://www.r-project.org). Supplementary Materials 4 and 5 show observed and forecasted GFRs for Brazil for the 13 selected Brazilian states using 2019–2021 ARPEN datasets (Supplementary Material 4: http://cadernos.ensp.fiocruz.br/static//arquivo/suple00230621-4_9817.pdf) and 2019–2021 SINASC datasets (Supplementary Material 5: http://cadernos.ensp.fiocruz.br/static//arquivo/supl-e00230621-5_7655.pdf).

This study was conducted under Institutional Review Board from the University of Texas at Austin, United States (approval #2018–01-0055), and the Brazilian National Research Ethics Committee (CONEP; CAAE: 34032920.1.0000.5149).

## Results

**Figura 1:**
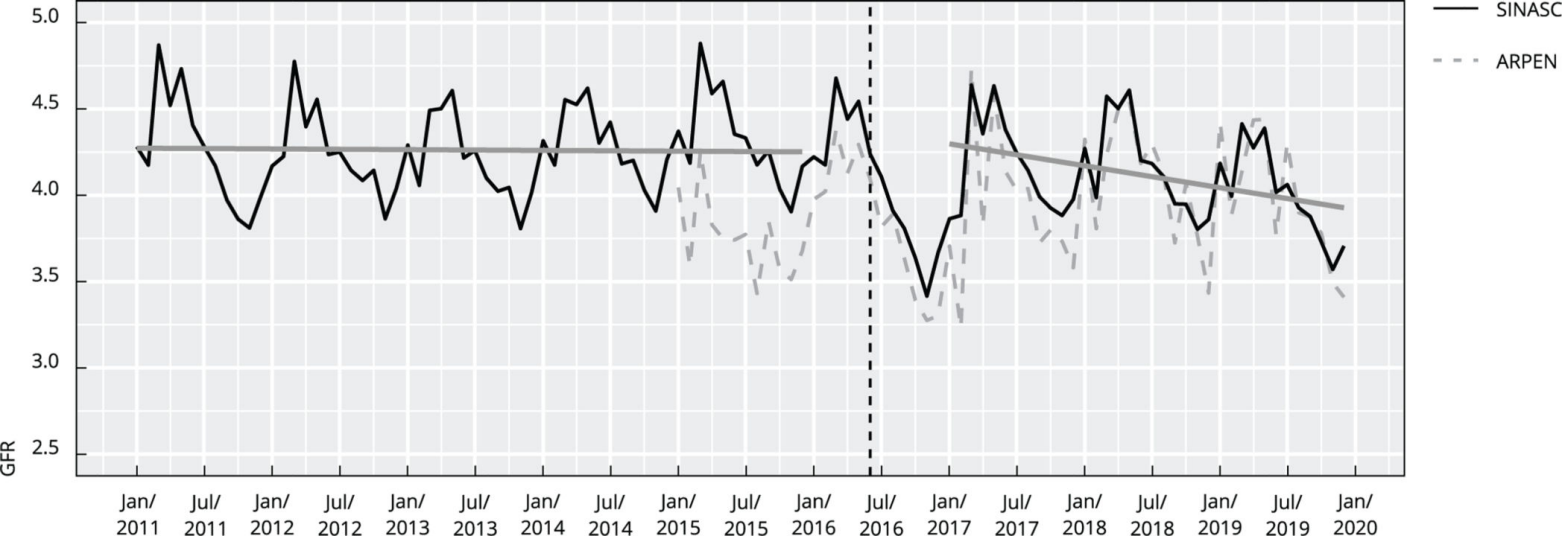
General fertility rate (GFR) by month. Brazilian Information System on Live Births (SINASC) and Association of Civil Registrar (ARPEN) datasets (observed), Brazil, 2011–2019.

The solid black line in Figure 1 shows GFRs using 2011–2019 SINASC data, while the dotted black line uses 2017–2021 ARPEN data to show the GFRs. We observe a remarkably steady trend in fertility in the period preceding the Zika epidemic (2011–2015), as solid gray line shows, with seasonal peaks from March to May. The time trend coefficient is not statistically different from zero.

This stable trend is disrupted in 2016 during the Zika epidemic, as previously documented ^3^. Figure 1 shows that fertility rates returned to pre-Zika levels in 2017, with no signs of a baby boom effect. In other words, the births that did not happen in 2016 were not replaced in 2017 or 2018. Instead, the months that were fertility “peaks” in 2018 and 2019 (March and May) show lower rates than peaks in previous years. Figure 1 shows a statistically significant trend of decline in fertility in the period 2017–2019 (regression time coefficient: −0.000348 GFR/month, CI: −0.000626; −0.000007).

**Figura 2:**
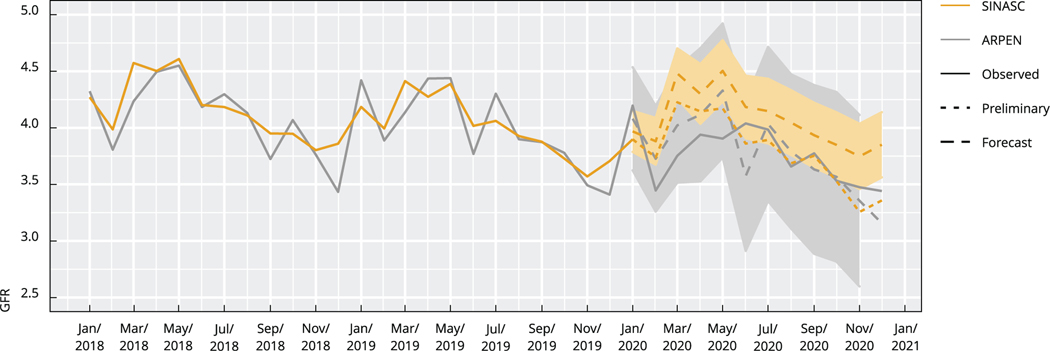
General fertility rate (GFR) by month. Brazilian Information System on Live Births (SINASC) and Association of Civil Registrar (ARPEN) datasets (observed and forecasted), selected states, Brazil, 2018–2020.

In Figure 2, we add ARIMA forecast rates (dotted lines) to the observed 2018–2019 SINASC rates (solid orange) and observed 2018–2020 ARPEN rates (solid grey). By comparing observed and forecasted ARPEN data for 2020 with forecasted SINASC data for 2020, we note that both datasets are consistent throughout 2020 for the 13 states, indicating that, since the 2020/2021 SINASC data are preliminary, it would be better to use ARPEN data to monitor fertility declines during 2021.

**Figura 3:**
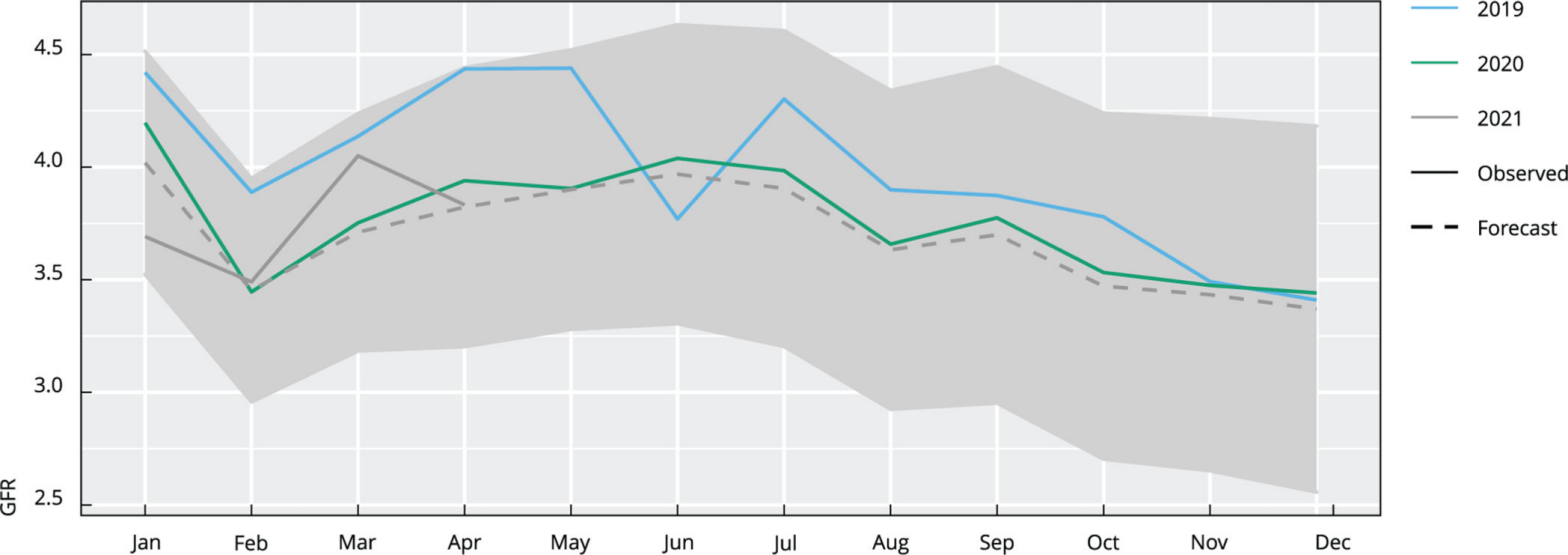
General fertility rates (GFRs) observed and ARIMA forecast. Association of Civil Registrar (ARPEN) dataset, Brazil, 2019–2021.

Regarding possible fertility changes driven by COVID-19, Figure 3 shows comparisons between observed and forecasted GFRs using ARPEN data (for states, Supplementary Material 4: http://cadernos.ensp.fiocruz.br/static//arquivo/supl-e00230621-4_9817.pdf). The results indicate that the observed rate was lower than the forecasted rate for January 2021. However, this difference is statistically insignificant and has decreased since February (and is still statistically insignificant). Because January 2021 was more than nine months after the initial surge of the coronavirus in Brazil in mid-March 2020, this difference suggests an important, yet short-lived, announcement effect. However, the magnitude of the January difference is heterogeneous across different states (Supplementary Material 4: http://cadernos.ensp.fiocruz.br/static//arquivo/supl-e00230621-4_9817.pdf): observed-forecasted differences are larger in the Southeast, where the first COVID-19 cases in Brazil were confirmed. Notwithstanding, none of these differences are statistically significant, even in the states with the largest numbers of documented COVID-19 cases ^17,18^.

## Discussion

As researchers continue the urgent task of documenting the effects of the COVID-19 pandemic, it remains critical to monitor its demographic consequences beyond mortality. This is particularly imperative in places where recent epidemics have already been consequential. In Brazil, for example, the Zika epidemic had already affected fertility before the onset of the COVID-19 pandemic. Our findings show that fertility was already at an all-time low in Brazil, and was actively in decline, even before COVID-19.

In our study, we show that fertility had recovered to pre-Zika levels in 2017, but that fertility had started to decline again shortly thereafter. This decline accelerated in 2019, a year before the first case of COVID-19 was reported in Brazil. We also show that the 2020–2021 differences between forecasted and observed fertility rates are large, yet statistically insignificant, even in states with high rates of COVID-19. Nevertheless, an aggregate-level analysis may mask the diversity of behavior among social groups that have responded differently to the epidemics. For example, a study has shown how fertility rates have responded to Zika epidemic along sociodemographic lines ^3^. Further studies using finalized 2020–2021 SINASC data should stratify rates by social groups, such as age, education level, socioeconomic status, and urban/rural residence. Although we only discussed ARPEN data from 2021, we show the projected decline for SINASC 2021 in Supplementary Material 5 (http://cadernos.ensp.fiocruz.br/static//arquivo/supl-e00230621-5_7655.pdf) to aid future research looking to compare observed and projected data once those data are finalized.

Considering how the spread of COVID-19 throughout Brazil has accelerated in 2021, we argue the uncertainties of the COVID-19 crisis have the potential to cause formidable compounding effects on fertility in Brazil. This is due to the back-to-back timing of the Zika virus and COVID-19 out-breaks, which gave women insufficient time to recalibrate their reproductive plans after the former epidemic before having to confront a new one. The pandemic also overlapped with acute economic and political crises, which are also likely to have exacerbated the low fertility trends that we found. Although it is difficult to identify the precise mechanisms driving fertility responses during exogenous crises, it is clear that the effects go beyond the direct effects of virus mortality. Epidemics can also affect fertility by inducing behavioral changes due to generalized uncertainty as a result of interruptions to health care access and childcare provision.

Finally, we also argue for the availability and dissemination of demographic data to the research and policy communities, even going beyond pre-existing political agreements. Public health interventions and policies during public health emergencies should focus on further understanding demographic trends besides mortality by the timely use of up-to-date data. Such policies should also aim to alleviate the structural causes of disparities in fertility intentions and behaviors to foster reproductive equity. Promoting policies that aim to provide women with the conditions to match their fertility behaviors to their fertility intentions becomes even more important in periods of public health crisis, when resources are shifted from reproductive care to confronting the outbreaks of novel diseases. For such crises disproportionately affect vulnerable populations, it is also important to further disentangle these patterns and focus on policies that target these groups. How these patterns unfold will reveal the long reach that these successive outbreaks will have on the number of children that women have in Brazil, and when they have them.

## Supplementary Material

Box S1

Figure S3

Figure S1

Figure S4

Figure S2

## References

[1] Johns Hopkins Coronavirus Resource Center. Brazil – COVID-19 overview. https://coronavirus.jhu.edu/region/brazil (accessed on 26/Nov/2021).

[2] Diaz-Quijano FA, Pelissari DM, Chiavegatto Filho ADP. Zika-associated microcephaly epidemic and birth rate reduction in Brazilian cities. Am J Public Health 2018; 108:514–6. 29470110 10.2105/AJPH.2017.304260 PMC5844397

[3] Marteleto LJ, Guedes G, Coutinho RZ, Weitzman A. Live births and fertility amid the Zika epidemic in Brazil. Demography 2020; 57:843–72. 32399856 10.1007/s13524-020-00871-x PMC7334083

[4] Centers for Disease Control and Prevention. CDC updates, expands list of people at risk of severe COVID-19 illness. https://www.cdc.gov/media/releases/2020/p0625-update-expands-covid-19.html (accessedon26/Nov/2021).

[5] Khalil A, von Dadelszen P, Draycott T, Ugwumadu A, O’Brien P, Magee L. Change in the incidence of stillbirth and preterm delivery during the COVID-19 pandemic. JAMA 2020; 324:705–6. 32648892 10.1001/jama.2020.12746 PMC7435343

[6] Souza ASR, Amorim MMR. Maternal mortality by COVID-19 in Brazil. Rev Bras Saúde Mater Infant 2021; 21:253–6.

[7] Villar J, Ariff S, Gunier RB, Thiruvengadam R, Rauch S, Kholin A, Maternal and neonatal morbidity and mortality among pregnant women with and without COVID-19 Infection: The INTERCOVID Multinational Cohort Study. JAMA Pediatrics 2021; 175:817–26. 33885740 10.1001/jamapediatrics.2021.1050 PMC8063132

[8] Coutinho RZ, Lima LC, Leocádio VA, Bernardes T. Considerações sobre a pandemia de COVID-19 e seus efeitos sobre a fecundidade e a saúde sexual e reprodutiva das brasileiras. Rev Bras Estud Popul 2020; 37:e0130.

[9] Marteleto LJ, Dondero M. Navigating women’s reproductive health and childbearing during public health crises: COVID-19 and Zika in Brazil. World Dev 2021; 139:105305. 10.1016/j.worlddev.2020.105305 PMC9053522 35495087

[10] Freire FHM, Gonzaga MR, Gomes MMF. Projeções populacionais por sexo e idade para pequenas áreas no Brasil. Revista Latinoamericana de Población 2019; 14:124–49.

[11] Departamento de Informática do SUS. Sistema de Informações sobre Nascidos Vivos. http://www2.datasus.gov.br/DATASUS/index.php?area=0205&id=6936&VObj=http://tabnet.datasus.gov.br/cgi/deftohtm.exe?sinasc/cnv/nv (accessed on 22/Sep/2021).

[12] Portal da Transparência. Registros. https://transparencia.registrocivil.org.br/registros (accessed on 22/Sep/2021).

[13] França EB, Ishitani LH, Teixeira RA, Abreu DMX, Corrêa PRL, Marinho F, Óbitos por COVID-19 no Brasil: quantos e quais estamos identificando? Rev Bras Epidemiol 2020; 23:E200053. 10.1590/1980-549720200053 32578810

[14] Lima EEC, Gonzaga MR, Freire FHM, Queiroz BL. Alternative information sources on deaths in Brazil in the context of the COVID-19 pandemic. Ottawa: Centre of Excellence for Civil Registration and Vital Statistics Systems; 2021.

[15] Neusser K. Time series econometrics. Cham: Springer International Publishing; 2016.

[16] Hyndman RJ, Khandakar Y. Automatic time series forecasting: the forecast package for R. J Stat Softw 2008; 27:1–22.

[17] Castro MC, Gurzenda S, Turra CM, Kim S, Andrasfay T, Goldman N. Reduction in life expectancy in Brazil after COVID-19. Nat Med 2021; 27:1629–35. 34188224 10.1038/s41591-021-01437-z PMC8446334

[18] Castro MC, Kim S, Barberia L, Ribeiro AF, Gurzenda S, Ribeiro KB, Spatiotemporal pattern of COVID-19 spread in Brazil. Science 2021; 372:821–6. 33853971 10.1126/science.abh1558

