# Supplementary material for: Fertility trends during successive novel infectious disease outbreaks: Zika and COVID-19 in Brazil
Source: Cad Saude Publica. Author manuscript; Available in PMC 2022 Dec 12. (PMC9744098; doi:10.1590/0102-311XEN230621)
Supplement: Box S1 [file NIHMS1845666-supplement-Box_S1.pdf]

Box S1 Auxiliary regressions results by state and dataset. Brazilian Information System on Live Births (SINASC) and Association of Civil Registrar (ARPEN) datasets, Brazil, 2017-2019.

FEDERATION UNIT	ADF TESTS FOR LIVE BIRTHS				EXPLANATORY POWER			RESIDUALS				CRITERIA
	SINASC	ARPEN	SINASC (*diff*)	ARPEN (*diff*)	α (p-value)	γ (p-value)	R^2	ADF	Mean	Lagged p-values	DW (p-value)	
RO	**0.0100**	**0.9541**	**0.0100**	**0.0100**	**0.7800**	**0.0000**	**0.6321**	**0.0100**	**0.0000**	**0.1589**	**0.1473**	**1**
AC	0.0219	0.0186	0.0100	0.0100	0.9861	0.0010	0.4120	0.0100	0.0000	0.0018	0.0012	0
AM	0.0373	0.0998	0.0100	0.0100	0.4949	0.1799	0.3126	0.0142	0.0000	0.6281	0.6977	0
RR	0.2156	0.0100	0.0100	0.0100	0.5309	0.5376	0.0154	0.0100	0.0000	0.0240	0.0270	0
PA	0.0212	0.6967	0.0100	0.0100	0.7436	0.0071	0.4765	0.0100	0.0000	0.4955	0.7509	0
AP	0.2075	0.0100	0.0350	0.0100	0.4128	0.4009	0.2377	0.0378	0.0000	0.1418	0.3266	0
TO	**0.0100**	**0.1028**	**0.0100**	**0.0100**	**0.7941**	**0.0000**	**0.5597**	**0.0100**	**0.0000**	**0.3622**	**0.3442**	**1**
MA	0.0100	0.4963	0.0100	0.0100	0.4194	0.1297	0.3734	0.0100	0.0000	0.7868	0.5115	0
PI	0.0100	0.5055	0.0100	0.0100	0.4838	0.3639	0.2832	0.0124	0.0000	0.5013	0.2565	0
CE	0.0137	0.2828	0.0126	0.0100	0.9592	0.0008	0.4690	0.0100	0.0000	0.5631	0.6269	0
RN	0.2383	0.2459	0.0100	0.0100	0.8188	0.0008	0.4791	0.0100	0.0000	0.2169	0.5224	0
PB	**0.0496**	**0.1130**	**0.0109**	**0.0100**	**0.8861**	**0.0000**	**0.6403**	**0.0100**	**0.0000**	**0.0716**	**0.0748**	**1**
PE	**0.0942**	**0.0465**	**0.0217**	**0.0100**	**0.8887**	**0.0000**	**0.6110**	**0.0100**	**0.0000**	**0.1845**	**0.2267**	**1**
AL	**0.3068**	**0.0100**	**0.0156**	**0.0100**	**0.8834**	**0.0001**	**0.5818**	**0.0100**	**0.0000**	**0.5849**	**0.8798**	**1**
SE	0.1661	0.1505	0.0406	0.0100	0.9541	0.0083	0.3786	0.0100	0.0000	0.6859	0.7264	0
BA	**0.4280**	**0.4129**	**0.0459**	**0.0100**	**0.5578**	**0.0000**	**0.5950**	**0.0151**	**0.0000**	**0.2988**	**0.2996**	**1**
MG	**0.3634**	**0.0894**	**0.0249**	**0.0100**	**0.7503**	**0.0000**	**0.6077**	**0.0100**	**0.0000**	**0.1764**	**0.1982**	**1**
ES	0.3823	0.0521	0.0203	0.0100	0.6863	0.0000	0.6315	0.0100	0.0000	0.0070	0.0042	0
RJ	**0.2510**	**0.1691**	**0.0422**	**0.0100**	**0.5576**	**0.0000**	**0.7539**	**0.0100**	**0.0000**	**0.1908**	**0.3345**	**1**
SP	**0.3301**	**0.1176**	**0.0371**	**0.0100**	**0.6604**	**0.0000**	**0.6926**	**0.0256**	**0.0000**	**0.1027**	**0.1487**	**1**
PR	0.3366	0.0866	0.0269	0.0100	0.6694	0.0000	0.7327	0.0100	0.0000	0.0161	0.0123	0
SC	0.2712	0.2368	0.0100	0.0100	0.9964	0.0005	0.4874	0.0100	0.0000	0.0818	0.1953	0
RS	**0.3406**	**0.4325**	**0.0100**	**0.0100**	**0.5932**	**0.0000**	**0.6248**	**0.0100**	**0.0000**	**0.0945**	**0.1447**	**1**
MS	**0.2684**	**0.2199**	**0.0503**	**0.0100**	**0.8861**	**0.0000**	**0.6852**	**0.0116**	**0.0000**	**0.3370**	**0.5872**	**1**
MT	**0.0427**	**0.4872**	**0.0100**	**0.0100**	**0.7679**	**0.0000**	**0.5662**	**0.0100**	**0.0000**	**0.1971**	**0.2331**	**1**
GO	**0.0974**	**0.1659**	**0.0100**	**0.0100**	**0.8669**	**0.0000**	**0.6165**	**0.0100**	**0.0000**	**0.0793**	**0.0888**	**1**
DF	0.2656	0.0100	0.0100	0.0100	0.5278	0.0000	0.7370	0.0271	0.0000	0.0023	0.0016	0

ADF: Augmented Dickey Fuller test; DW: Durbin-Watson test.

Source: Information System on Live Births (SINASC) and Association of Civil Registrar (ARPEN).
