# Supplementary material for: Fertility trends during successive novel infectious disease outbreaks: Zika and COVID-19 in Brazil
Source: Cad Saude Publica. Author manuscript; Available in PMC 2022 Dec 12. (PMC9744098; doi:10.1590/0102-311XEN230621)
Supplement: Figure S3 [file NIHMS1845666-supplement-Figure_S3.pdf]

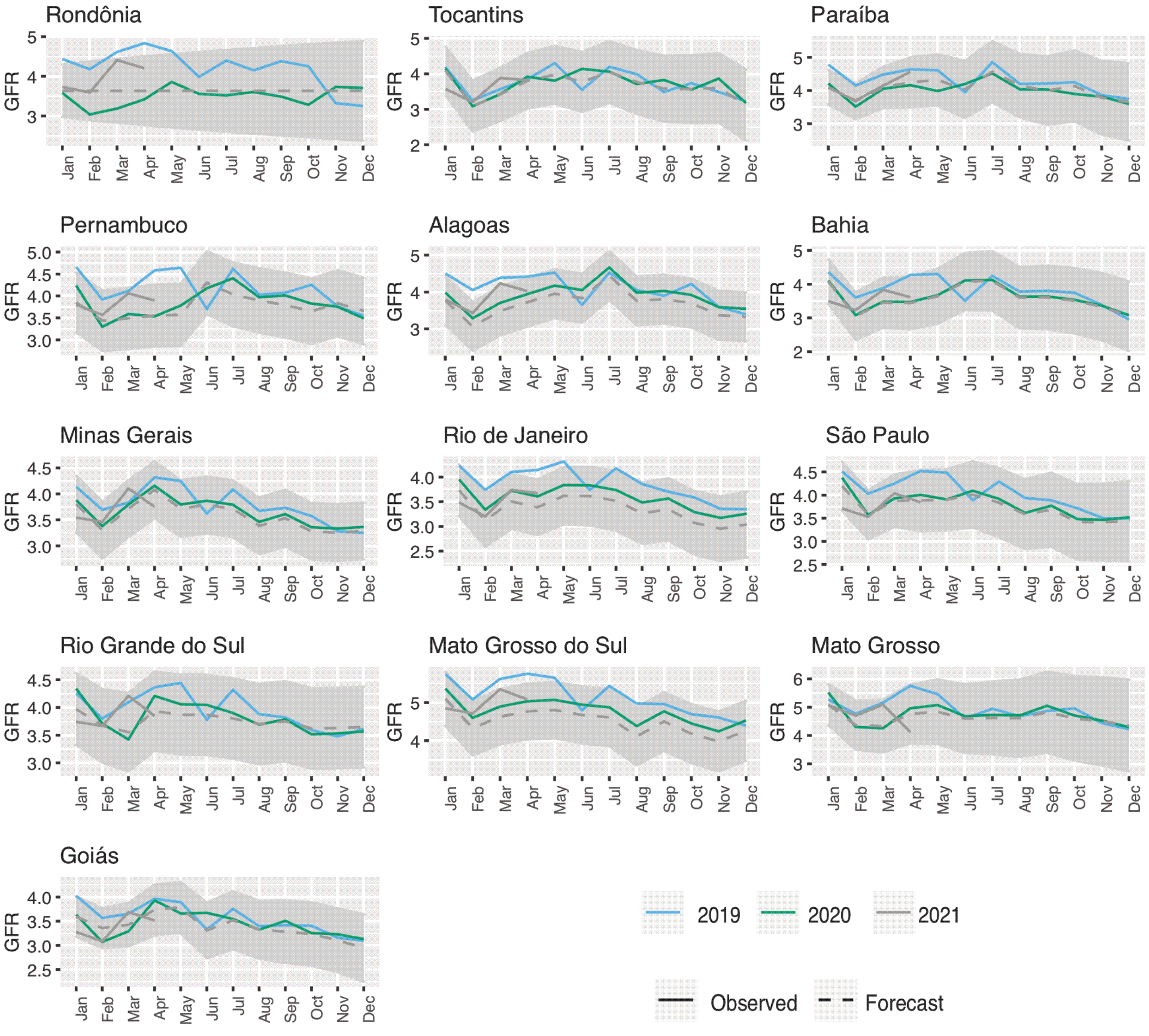

Source: SINASC (Ministério da Saúde 2021); ARPEN (Associação Nacional dos Registradores de Pessoas Naturais 2021)

Figure S3 General fertility rates (GFRs) observed and ARIMA forecast. Association of Civil Registrar (ARPEN) datasets, selected states, 2019-2021.
