# Supplementary material for: Fertility trends during successive novel infectious disease outbreaks: Zika and COVID-19 in Brazil
Source: Cad Saude Publica. Author manuscript; Available in PMC 2022 Dec 12. (PMC9744098; doi:10.1590/0102-311XEN230621)
Supplement: Figure S1 [file NIHMS1845666-supplement-Figure_S1.pdf]

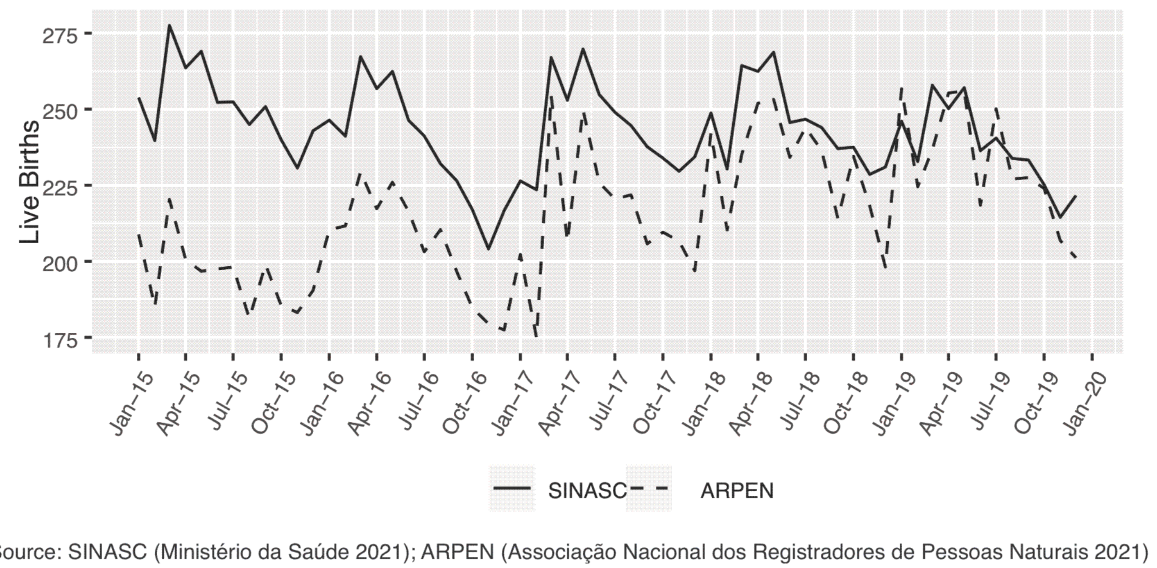

Figure S1 Live births (thousand). Brazilian Information System on Live Births (SINASC) and Association of Civil Registrar (ARPEN) datasets, Brazil, 2015-2019.
