# Supplementary material for: Fertility trends during successive novel infectious disease outbreaks: Zika and COVID-19 in Brazil
Source: Cad Saude Publica. Author manuscript; Available in PMC 2022 Dec 12. (PMC9744098; doi:10.1590/0102-311XEN230621)
Supplement: Figure S2 [file NIHMS1845666-supplement-Figure_S2.pdf]

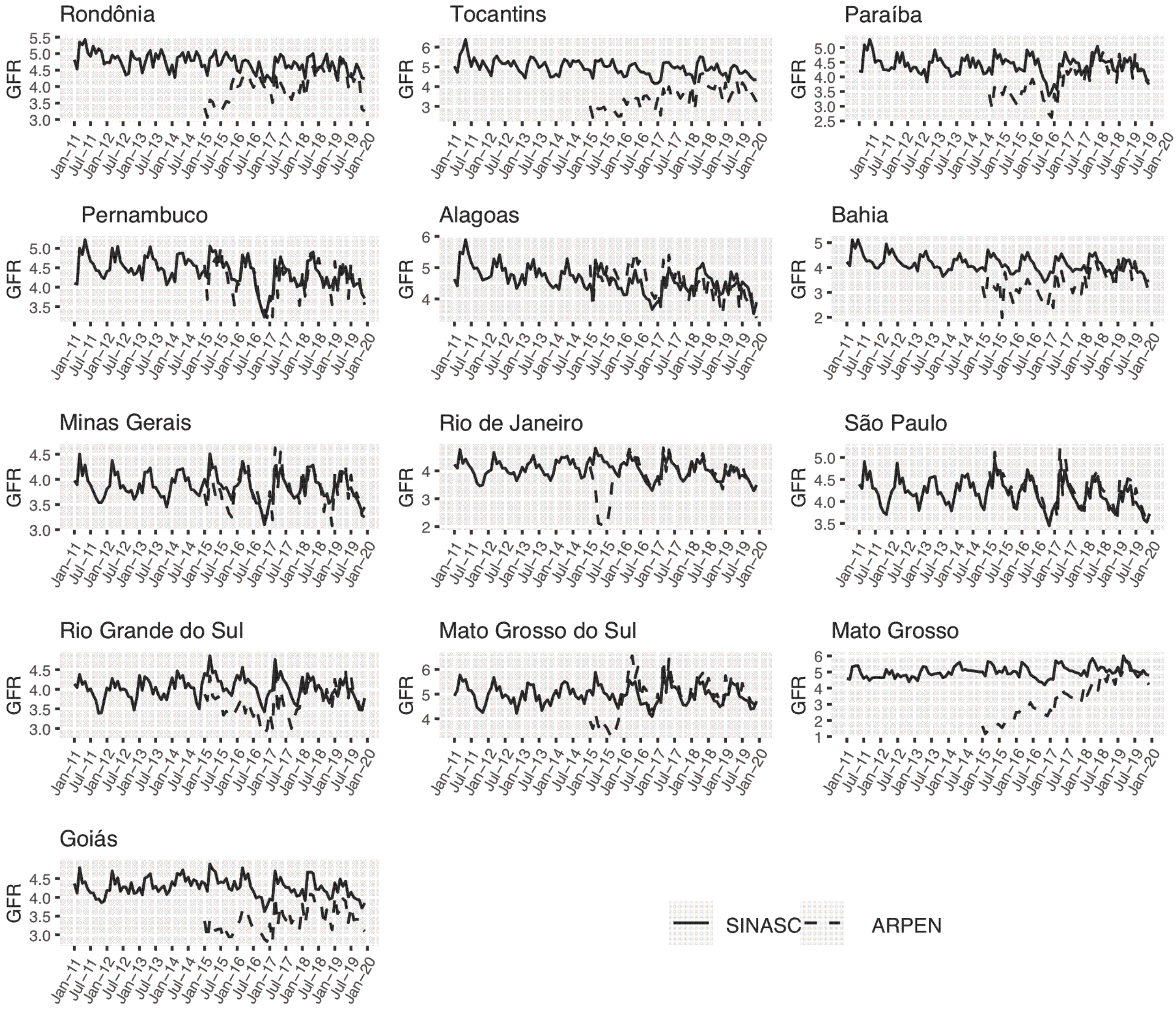

Source: SINASC (Ministério da Saúde 2021); ARPEN (Associação Nacional dos Registradores de Pessoas Naturais 2021)

Figure S2 General fertility rate (GFR) by month and selected states. Brazilian Information System on Live Births (SINASC) and Association of Civil Registrar (ARPEN) datasets (observed), Brazil, 2011-2019.
